# Metabolites of gut microbiome are associated with glucose metabolism in non-diabetic obese adults: a Chinese monozygotic twin study

**DOI:** 10.1186/s13098-021-00724-6

**Published:** 2021-10-09

**Authors:** Ke Yu, Cai-Guo Yu, Xing-Qi Yin, Zong-Wei Wang, Xiao-Bo Wang, Li-Li Wang, Shuang Guo, Ya-Xin An, Dong Zhao

**Affiliations:** 1 Center for Endocrine Metabolism and Immune Diseases, Beijing Luhe Hospital, Capital Medical University, Beijing, 101149 China; 2 Beijing Key Laboratory of Diabetes Research and Care, Beijing, 101149 China

**Keywords:** Gut microbiome, Glucose metabolism, Twin study, Obesity

## Abstract

**Background:**

Evidence suggests gut microbiome is associated with diabetes. However, it’s unclear whether the association remains in non-diabetic participants. A Chinese monozygotic twin study, in which the participants are without diabetes, and are not taking any medications, was conducted to explore the potential association.

**Methods:**

Nine pairs of adult monozygotic twins were enrolled and divided into two twin-pair groups (a and b). Clinical and laboratory measurements were conducted. Visceral adipose tissue (VAT) was assessed. Fecal samples were collected to analyze the microbiome composition by 16S rDNA gene amplicon sequencing. Liquid chromatography mass spectrometry was performed to detect the metabolites.

**Results:**

The participants aged 53 years old averagely, with 8 (88.9%) pairs were women. All the participants were obese with VAT higher than 100 cm^2^ (152.2 ± 31.6). There was no significant difference of VAT between the twin groups (153.6 ± 30.4 cm^2^ vs. 150.8 ± 29.5 cm^2^, p = 0.54). Other clinical measurements, including BMI, lipid profiles, fasting insulin and blood glucose, were also not significantly different between groups (p ≥ 0.056), whereas HbA1c level of group a is significantly higher than group b (5.8 ± 0.3% vs. 5.6 ± 0.2%, p = 0.008). The number and richness of OTUs are relatively higher in group a, and 13 metabolites were significantly different between two groups. Furthermore, several of the 13 metabolites could be significantly linked to special taxons. The potential pathway involved drug metabolism-other enzymes, Tryptophan metabolism and Citrate cycle.

**Conclusions:**

Gut microbiome composition and their metabolites may modulate glucose metabolism in obese adults without diabetes, through Tryptophan metabolism, Citrate cycle and other pathways.

## Background

Increasing evidence suggests the gut microbiome is associated with metabolic diseases, especially obesity and type 2 diabetes (T2D) [1–4]. However, the participants of most previous studies [5–10] are patients with type 2 diabetes or pre-diabetes, with different genetic background, and taking different kinds of anti-diabetic drugs, which may have confounding effects on the gut microbiome composition and fecal metabolites [11–13]. Thus, it’s unclear whether the association remains in healthy or early subclinical status without obvious confounding factors. Monozygotic twins shared the same genotype and early environmental exposures, and thus, potentially similar gut microbiome composition [14].

Only few work publications were reported to address the abovementioned issue in PubMed with keywords as “gut microbiome” and “Twin” [14–16] (Table 1), one of the which indicated that microbiome changes were associated with sub-clinical state of T2D in participants neither obese nor diabetic [15]. However, none of the three studies included Chinese participants. In this work, a Chinese monozygotic (MZ) twin study was conducted to explore the potential association of gut microbiome with glucose metabolism in healthy obese participants. Twins in the study grew up in the same family, without known diabetes, without taking antibiotics or other medications, to avoid other factors that may influence the gut microbiome.

**Table 1:** Twin studies of gut microbiome

Authors	Numbers of participants	Objects	Tests	Conclusions
Bowyer et al. (2019)	1163 healthy individuals (48 twin pairs discordant for Index of Multiple Deprivation)	Socioeconomic Status and the Gut Microbiome	16S rRNA microbiota data, and all considered co-variables.	The greater the difference in twin pair Index of Multiple Deprivation, the greater the dissimilarity of their microbiota.
Yassour et al. (2016)	20 monozygotic healthy Korean twins	Microbial changes during the sub-clinical state of T2D	Total DNAs from fecal samples, and clinical metadata variables	Changes in composition of the sub-clinical gut microbiome suggests a role prior to the onset of T2D, and functional changes reflects a response to oxidative stress.
Turnbaugh et al. (2009)	154 individuals (adult female monozygotic and dizygotic twin pairs concordant for leanness or obesity, and their mothers)	16 S rRNA sequences, plus 2.14 gigabases from their microbiomes	How host genotype, environmental exposure and host adiposity influence the gut microbiome	Obesity is associated with phylum-level changes in the microbiota, reduced bacterial diversity and altered representation of bacterial genes and metabolic pathways.

Microbial metabolites related with host glucose metabolism included Short-Chain Fatty Acids (SCFAs), such as acetate, butyrate, and propionate, which are the major end products of carbohydrates [17]. Besides SCFAs, branched-chain amino acids (BCAAs), bile acids, sulfur-containing amino acids, indole derivatives, trimethylamine N-oxide (TMAO) and vitamins were also involved in the regulation of insulin resistance [18]. In the present study, the above metabolites were tested in the fecal samples to detect the associations with glucose metabolism.

## Methods

### Study design

Nine pairs of adult MZ twins who were native residents in Tongzhou District of Beijing were enrolled in our study. The twin pairs grew up in the same family, without diagnosed diabetes, and not taking antibiotics or other medications that may influence the gut microbiome in the last two weeks before coming to hospital. The twin pairs were excluded as long as one of them is pregnant, or with tumor history, or with mental disease, or with recent history of diarrhea or intestinal infection.

The participants were divided into two twin-pair groups (a and b). Clinical and laboratory measurements were conducted. Visceral adipose tissue (VAT) was assessed. Fecal samples were collected to analyze the microbiome composition by 16S rDNA gene amplicon sequencing. Liquid chromatography mass spectrometry was performed to detect the metabolites.

### Clinical and laboratory measurements

The nurses first administered questionnaires, inquiring into each participant’s medical history, smoking and drinking habits, and intake of medications. Then, venous blood samples were obtained after 8 to 10 h of fasting. Blood samples were analyzed for serum levels of HbA1c, glucose, insulin, triglycerides, total low-density lipoprotein (LDL) and high-density lipoprotein (HDL) cholesterol, glutamic-pyruvic transaminase enzyme (ALT), glutamic-oxaloacetic aminotransferase(AST), gamma-glutamyltransferase (GGT), and serum creatinine. Physical examinations were also performed, including body weight, body height, waist and hip circumference. Visceral adipose fat (VAT) was evaluated for each participant (Inbody 770, Biospace Co. Ltd.).

Blood samples were also obtained and sent to Beijing Genomics Institute to extract DNAs and identify the egg type. Short tandem repeats were applied to identify and confirm the egg type.

### Microbiome composition analysis

Total genome DNA from fecal samples was extracted using Soil DNA Kit according to manufacturer’s protocols. DNA concentration was monitored by Qubit® dsDNA HS Assay Kit.

20–30ng DNAs were used to generate amplicons. V3 and V4 hypervariable regions of prokaryotic 16S rDNA were selected for generating amplicons and following taxonomy analysis. The concentration of DNA library was validated by Qubit3.0 Fluorometer. Quantify the library to 10 nM, DNA libraries were multiplexed and loaded on an Illumina MiSeq or NovaSeq instrument according to manufacturer’s instructions (Illumina, San Diego, CA, USA). Sequencing was performed using paired-end. Image analysis and base calling were conducted by the Control Software embedded in the instrument.

The effective sequences were used in final analysis. Sequences were grouped into operational taxonomic units (OTUs) using the clustering program VSEARCH (1.9.6) against the UNITE ITS database (https://unite.ut.ee/) pre-clustered at 97% sequence identity. The Ribosomal Database Program (RDP) classifier was used to assign taxonomic category to all OTUs at confidence threshold of 0.8. The RDP classifier used the UNITE ITS database which has taxonomic categories predicted to the species level.

### Fecal metabolites analysis

Various metabolites, including methanol, acetonitrile, 2-chlorophenylalanine, formic acid, ammonium formate and ddH_2_O, were detected based on liquid chromatography mass spectrometry (LC/MS) in fecal samples.

Fecal samples were thawed at 4 °C. 100 µL of each sample was transferred into 1.5 mL centrifuge tubes, and 400 µL of methanol (pre-cooled at − 20 °C) were added to each tube and vortex for 60 s. Then, the mixtures were centrifuged for 10 min at 12,000 rpm 4 °C and all supernatant in each tube was transferred into another 1.5 mL centrifuge tube, and samples were blow-dried by vacuum concentration. The processed supernatant was dissolved with 150 µL 2-chlorobenzalanine (4 ppm) methanol aqueous solution (4 °C), and filtered through a 0.22 μm membrane to obtain the prepared sample extracts for LC-MS.

Chromatographic separation was accomplished in an Thermo Ultimate 3000 system equipped with an ACQUITY UPLC® HSS T3 (150 × 2.1 mm, 1.8 μm, Waters) column maintained at 40 °C. The temperature of autosampler was 8 °C. Gradient elution of analytes was carried out with 0.1% formic acid in water (C) and 0.1% formic acid in acetonitrile (D) or 5 mM ammonium formate in water (A) and acetonitrile (B) at a flow rate of 0.25 mL/min. Injection of 2µL of each sample was done after equilibration. An increasing linear gradient of solvent B (v/v) was used as follows: 0–1 min, 2% B/D; 1–9 min, 2–50% B/D; 9–12 min, 50–98% B/D; 12–13.5 min, 98% B/D; 13.5–14 min, 98–2% B/D; 14–20 min, 2% D-positive model (14–17 min, 2% B-negative model).

The ESI-MSn experiments were performed on the Thermo Q Exactive Focus mass spectrometer with the spray voltage of 3.8 kV and − 2.5 kV in positive and negative modes, respectively. Sheath gas and auxiliary gas were set at 45 and 15 arbitrary units, respectively. The capillary temperature was 325 °C, respectively. The Orbitrap analyzer scanned over a mass range of m/z 81–1000 for full scan at a mass resolution of 70,000. Data dependent acquisition (DDA) MS/MS experiments were performed with HCD scan. The normalized collision energy was 30 eV. Dynamic exclusion was implemented to remove some unnecessary information in MS/MS spectra.

### Statistical analysis

Database management and statistical analysis were carried out using SAS 9.4 software (Cary, NC). The central tendency (spread) was represented by the arithmetic mean (SD). To compare means and proportions, paired t-test and the χ^2^-statistic were applied, respectively. Significance was a 2-tailed α-level of 0.05 or less.

## Results

### Characteristics and glucose levels of participants

Nine obese twin pairs without diabetes were enrolled in this study, aged 53 years old averagely, with 8 (88.9%) pairs were women. The twins were divided to two groups (a vs. b) for further analysis. All of the participants were obese with VAT higher than 100 cm^2^ (152.2 ± 31.6), and there was no difference between the twin groups (153.6 ± 30.4 cm^2^ vs. 150.8 ± 29.5 cm^2^, P = 0.54). However, HbA1c levels were significantly different, averaged 5.8 ± 0.3% vs. 5.6 ± 0.2% (P = 0.008). Fasting blood glucose and body mass index averaged 5.82 ± 0.71 vs. 5.58 ± 0.34 mmol/L (P = 0.29), 29.7 ± 3.4 vs. 28.8 ± 3.2 (P = 0.056), respectively. The other characteristics of participants were described in Table 2.

**Table 2:** Characteristics of participants

Characteristic	Different twin pairs		
	Group a	Group b	*P* value
Number	9	9	
Number (%) with characteristic			
Women	8 (88.9)	8 (88.9)	–
Current drinking	1 (11.1)	1 (11.1)	–
Current smoking	1 (11.1)	1 (11.1)	–
Mean of characteristic			
Age, years	53.0 ± 8.6	53.0 ± 8.6	–
Body mass index, kg/m^2^	29.7 ± 3.4	28.8 ± 3.2	0.056
Visceral fat, cm^2^	153.6 ± 30.4	150.8 ± 29.5	0.54
HbA1c, %	5.8 ± 0.3	5.6 ± 0.2	0.008†
Plasma glucose, mmol/L	5.82 ± 0.71	5.58 ± 0.34	0.29
Serum insulin, mU/L	11.7 ± 4.4	12.0 ± 3.0	0.77
Triglyceride, mmol/L	1.66 ± 0.63	1.53 ± 0.54	0.60
Total cholesterol, mmol/L	5.15 ± 1.22	4.21 ± 0.97	0.16
HDL cholesterol, mmol/L	1.25 ± 0.23	1.23 ± 0.11	0.76
LDL cholesterol, mmol/L	3.34 ± 0.96	2.90 ± 0.50	0.27
ALT, U/L	24.9 ± 11.1	21.8 ± 10.2	0.074
AST, U/L	20.6 ± 7.6	20.1 ± 11.4	0.82
GGT, U/L	21.9 ± 8.8	20.3 ± 8.4	0.13
Serum creatinine, µmol/L	63.0 ± 8.2	64.8 ± 9.1	0.21

*HDL* high-density lipoprotein

For continuously distributed variables, reported values are arithmetic (±SD).  Significance of the difference with the adjacent left column: *P ≤ 0.05; ^†^*P *≤ 0.01

### Differences of gut microbiome composition

**Fig. 1:**
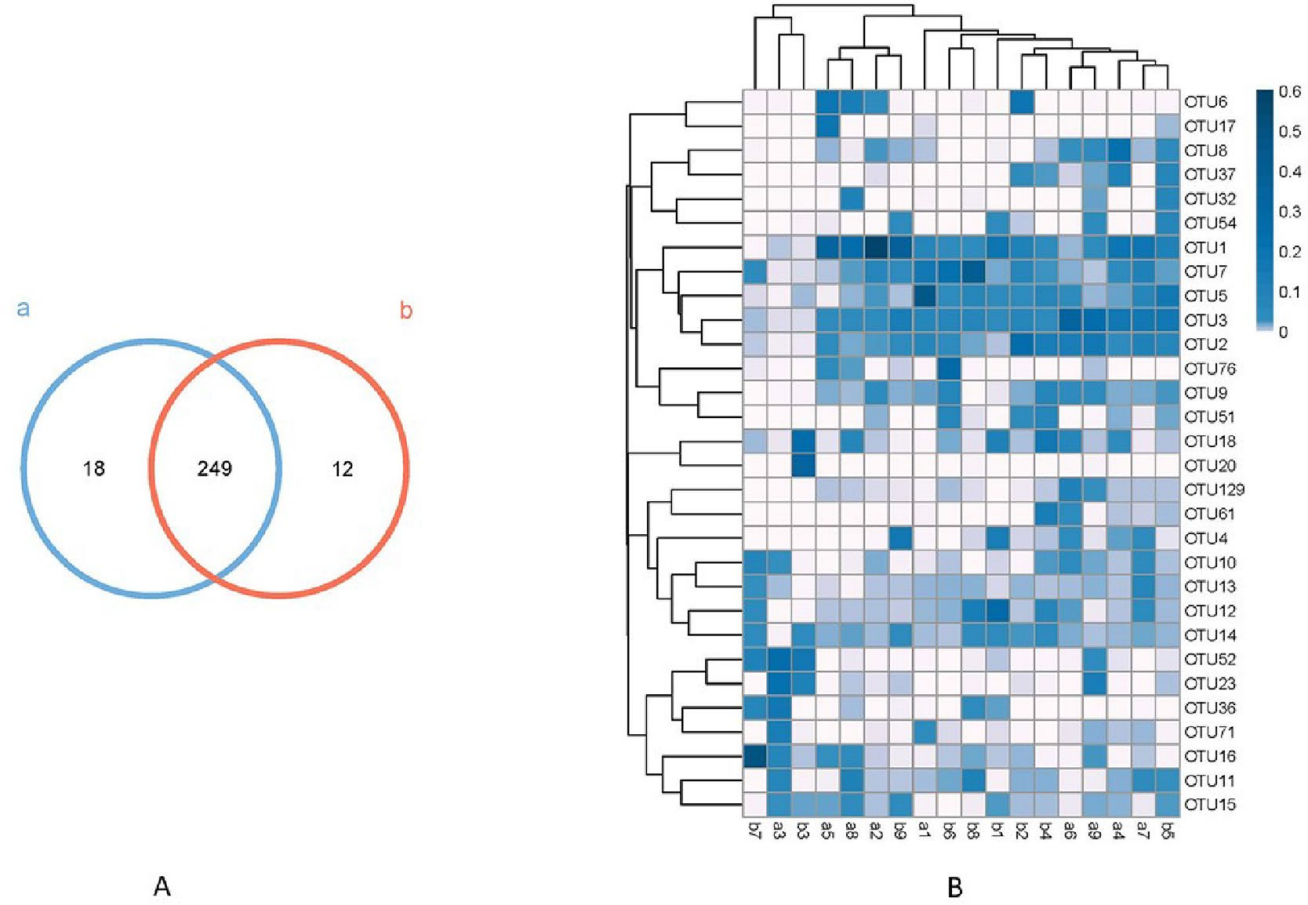
OTUs in groups and participants. In **A**, different colors of circles represent different group, and the numbers represent the shared and unique OTUs for two groups. In **B**, 30 OTUs with the highest richness in each participant were displayed in the clustered heatmap. The depths of colors representing the richness of OTUs. The row name is OUT ID, and the column name represents each participant in different twin groups

Two hundred and seventy-nine OTUs were detected in all samples. The twin groups shared 249 same OTUs, with 18 unique OTUs for group a and 12 for group b (Fig. 1A). Thirty OTUs with the highest richness in each participant were displayed in the clustered heatmap, with the depths of colors representing the richness (Fig. 1B). The corresponding top five taxons were k__Bacteria, p__Firmicutes, c__Clostridia, o__Clostridiales and f__Lachnospiraceae.

### Differences of fecal metabolite profiles

**Fig. 2:**
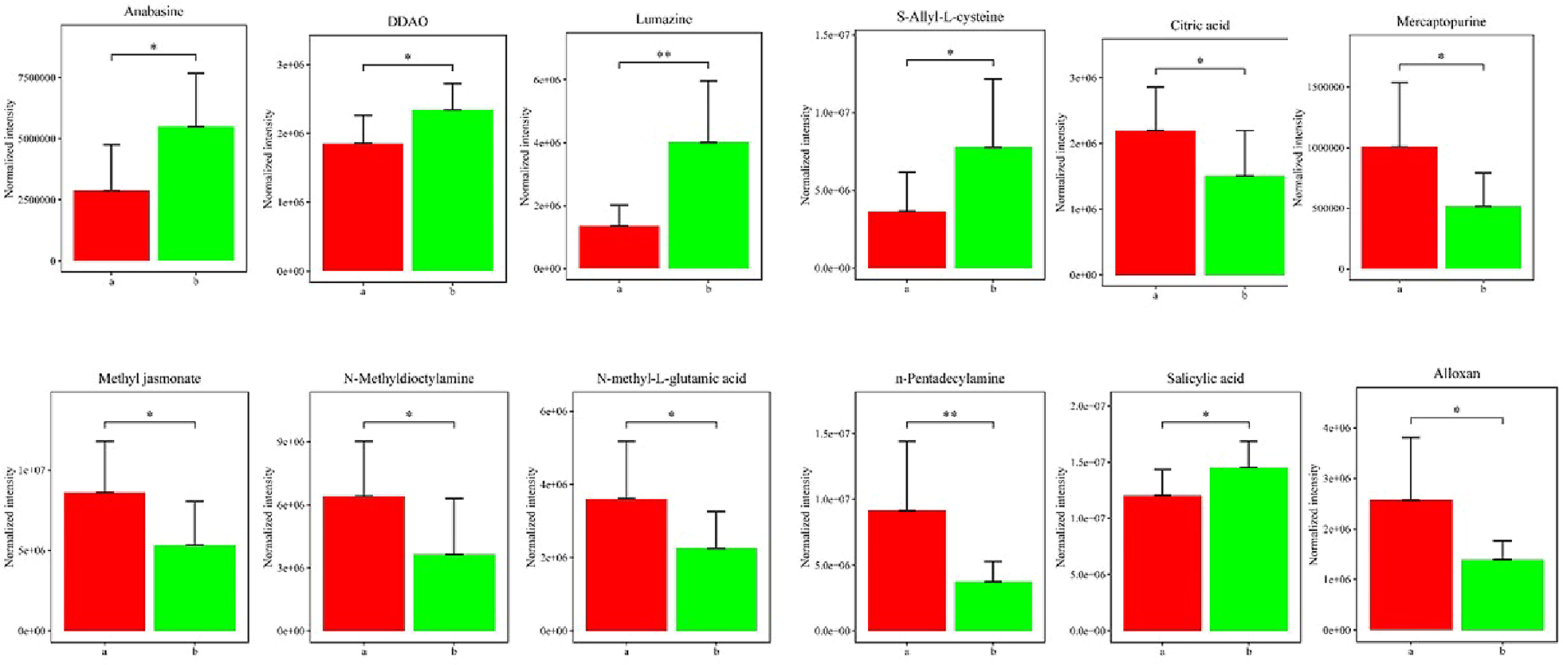
Fecal metabolites with significantly differences between groups ( a vs. b). *P ≤ 0.05

Thirteen metabolites were significantly different between two groups (Fig. 2), which includes anabasine, DDAO, lumazine, *S*-Allyl-l-cysteine, citric acid, alloxan, indoleacetic acid, mercaptopurine, Methyl Jasmonate, *N*-methyl-l-glutamic acid, *N*-Methyldioctylamine, *n*-Pentadecylamine, and Salicylic Acid. The former four metabolites were significantly lower in group a, while the others were significantly higher.

Correlation analysis were performed between the above 13 metabolites with Pearson Correlation Coefficient. Nineteen significant correlations were detected between each two metabolites. Four significant correlations were detected for *n*-Pentadecylamine, three for alloxan, anabasine and DDAO and two for mercaptopurine (Fig. 2).

**Fig. 3:**
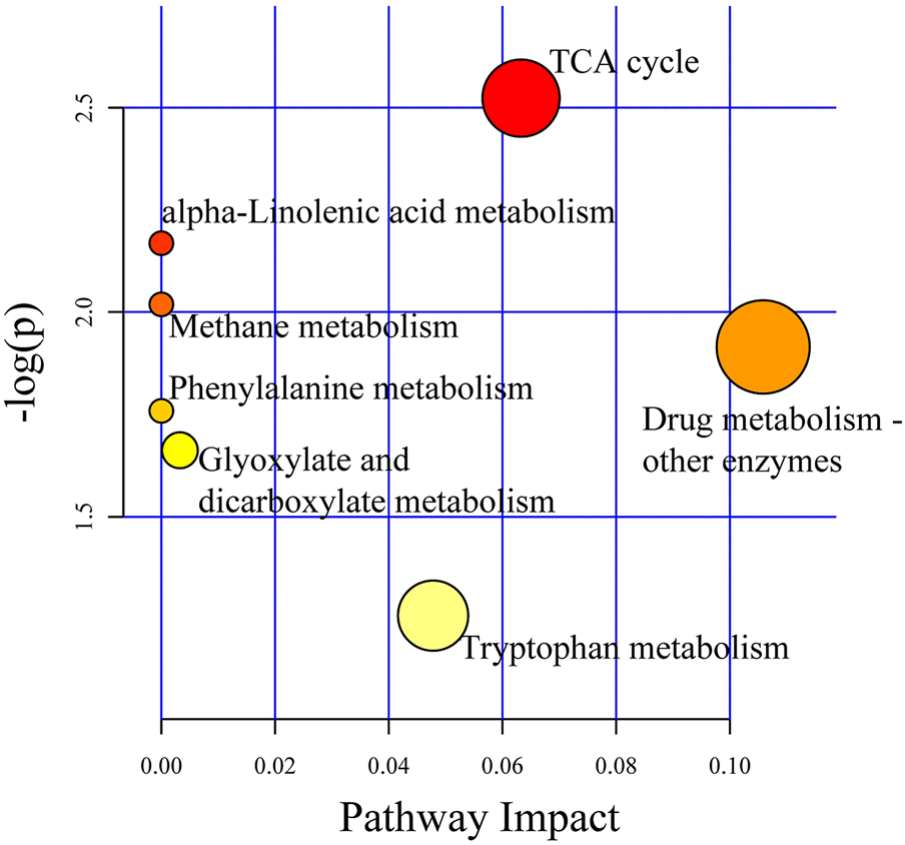
Pathway impact factors of seven potential metabolic pathways. The X-axis is the impact factor for each pathway, and the Y-axis is the log transformation of P value

Pathway impacts were also performed. Seven potential pathways were found related with gut microbiome and the metabolites. The three pathways with greatest impacts were Drug metabolism, Tryptophan metabolism and Citrate cycle, with impacts from 0.048 to 0.11 (Fig. 3).

### Correlation analysis of gut microbiome and metabolites

**Fig. 4:**
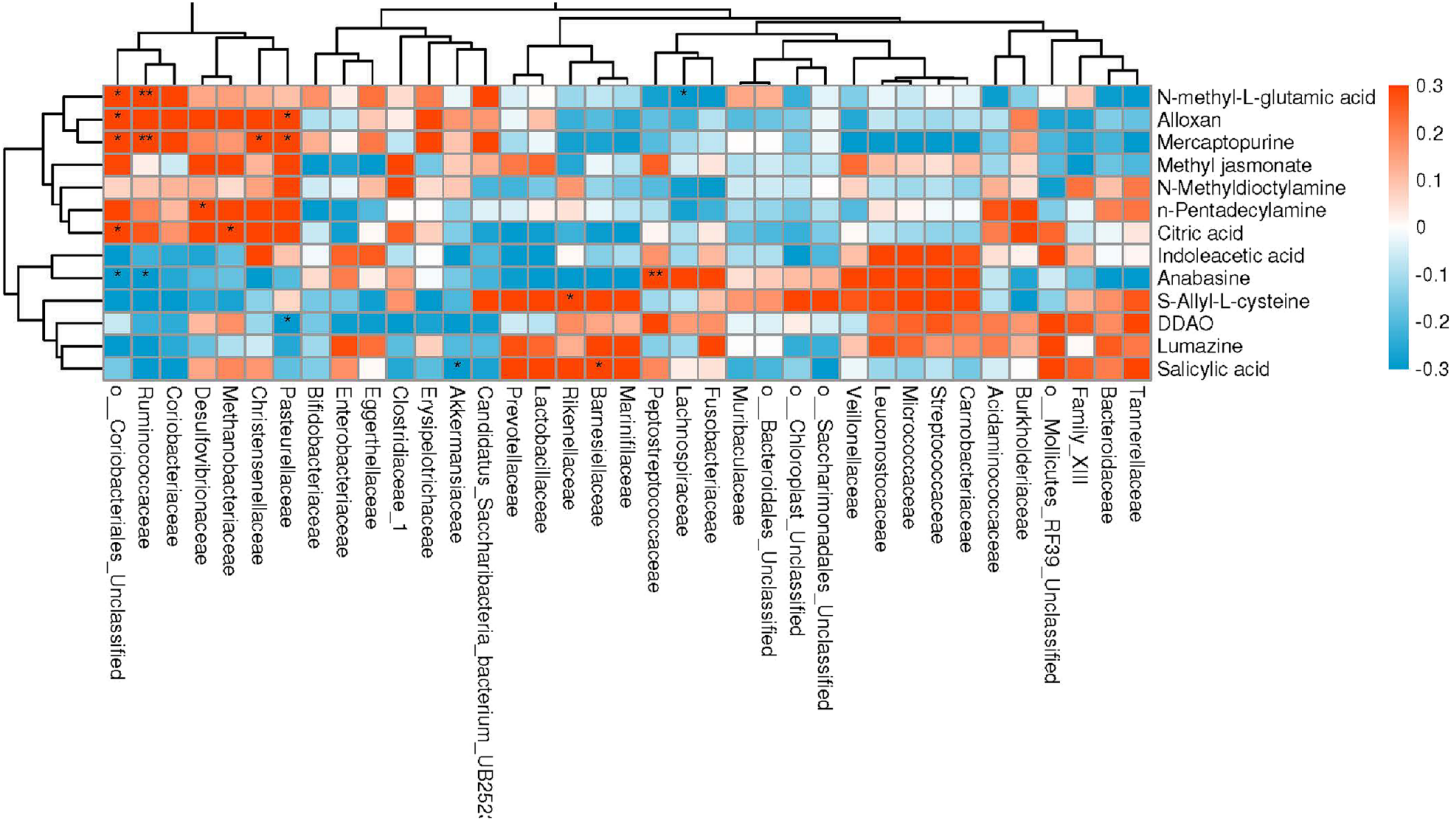
Correlation analysis of metabolites and taxons at family levels. The X-axis is the different families of microbiome taxons, the Y-axis is the different metabolites. Asterisks in the boxes represents the significant differences between specific metabolites and microbiome families. Five metabolites (*N*-methyl-l-glutamic acid, Alloxan, Mercaptopurine, Citric acid and Anabasine) were found significant for o__Coriobacteriales_Unclassified family. Mercaptopurine was significantly different in four families. *P ≤ 0.05, **P ≤ 0.01

Correlation analysis were performed to explore the potential associations of significantly different metabolites with special taxons. Twenty significant associations were found at family levels, with five metabolites (*N*-methyl-l-glutamic acid, Alloxan, Mercaptopurine, Citric acid and Anabasine) significant for o__Coriobacteriales_Unclassified family, and Mercaptopurine was the only metabolite that was significantly different in four families (Fig. 4).

## Discussion

This study aimed to explore whether the gut microbiome composition and the metabolites were associated with the glucose metabolism in nine obese MZ twin pairs without history of diabetes. The twins were divided into group a and group b. There were no significant differences for VAT or BMI or other characteristic between two groups, whereas the level of HbA1c is significantly higher in group a. 16S rDNA-based high-throughput sequencing and LC/MS were performed in 18 fecal samples from these nine MZ twin pairs. Analysis showed that the number and richness of OTUs are relatively higher in group a, and 13 metabolites were significantly different between two groups. Furthermore, several of the 13 metabolites were significantly associated with special taxons, which indicated that the gut microbiome may modulate the glucose metabolism through the gut microbiome composition and their metabolites. The potential pathways involved Drug metabolism, Tryptophan metabolism and Citrate cycle.

Previous studies performed in diabetic patients with different ethnicities mostly suggested the gut microbiome composition changes are associated with the onset or the development of the disease. However, the differential communities or taxons are inconsistent. Three Chinese studies were conducted in participants at different stages of glucose intolerance status: normal glucose tolerance (NGT), prediabetes (Pre-DM), and T2DM patients. Zhang et al. [5] analyzed 121 participants: 44 NGTs, 64 Pre-DMs and 13 newly diagnosed T2DM. Results showed that Verrucomicrobiae had a significantly lower abundance in both the pre-DM and T2DM groups. Zhao et al. [8] performed analysis in T2DM patients with or without complications, and the healthy controls. Results suggested higher abundance of Proteobacteria and higher ratio of Firmicutes/Bacteroidetes in T2DM patients. Zhong et al. [7] explored the gut metagenomics and metaproteomics signatures in Pre-DMs, T2DMs without treatment and NGTs. They found a significantly higher abundance of Megasphaera elsdenii (MLG-1568) in both T2DMs and Pre-DMs than in NGTs. A recent African study [10] also revealed that gut microbiota composition is associated with T2DM, with identification of higher richness of Desulfovibrio piger, Prevotella, Peptostreptococcus, and Eubacterium in T2DM group. Bhute et al. [6] assessed gut microbial diversity in 49 Indian participants who were also divided into three groups: New-DMs, Known-DMs and healthy participants. Results indicated that microbial dysbiosis may not be just limited to eubacteria in diabetes, which may also extend into other two domains leading to trans-domain dysbiosis in microbiota. Another recent study conducted by Gaike et al. [9] suggested that gut microbial diversity of newly diagnosed T2DMs is significantly different from that of NGTs, whereas this difference was not observed between the Pre-DM group and NGT group. The inconsistent microbiota composition results from these above studies indicate the complexity of gut microbiome changes in human participants. On the other hand, it may also indicate the potential interference of various confounding factors, such as the genotype, the different environment exposures and the ethnicities.

A Korean MZ twin study [15] conducted gut microbiome analysis in 20 MZ twins neither obese nor diabetic, with 36 fecal samples collected. They analyzed the association of changes in microbiome composition with different factors, such as BMI and glucose levels. Results suggested the decrease in Akkermansia muciniphila may occur prior to the onset of diabetes, and strain-level differences in composition were observed despite of species-level similarities in the twin pairs.

The potential mechanisms of the gut microbiota and glucose metabolism mainly involve several metabolites [18], including beneficial metabolites, such as short-chain fatty acids (SCFAs), sulfur-containing amino acids, bile acids, and indole derivatives, and also potentially harmful metabolites, such as branched-chain amino acids(BCAAs) and lipopolysaccharide (LPS).

In the current study, anabasine, DDAO, lumazine, *S*-Allyl-l-cysteine were identified to be lower, while citric acid, alloxan, indoleacetic acid, mercaptopurine, methyl jasmonate, *N*-methyl-l-glutamic acid, *N*-Methyldioctylamine, *n*-Pentadecylamine, and Salicylic acid were higher in group a with higher HbA1c levels. And some of the above metabolites could be linked to special taxons, which indicated that the impaired glucose metabolism may be associated with gut micorbiome composition and their metabolites.

In comparison with previous studies, the present study was performed in MZ twins. The participants shared same genetic background and growth environment, and none of them was taking any medications when enrolled in this study, which can eliminate the effect of medications on gut microbiome. Thus, the design of our study is more favorable to identify the unique microbial changes and to explore the potential taxons and their metabolites associated with the development or prevention of impaired glucose metabolism. Nevertheless, reported findings must be interpreted within the context of their limitations. First, the sample size is relatively small. Second, most of the participants are female, the gender bias cannot be excluded. Third, the study design is cross-sectional, which cannot make causal explanations.

## Conclusions

Results of the study indicate that higher levels of HbA1c in the twin pairs may be associated with some metabolites of the gut microbiome, including anabasine, DDAO, lumazine, *S*-Allyl-l-cysteine, citric acid, alloxan, indoleacetic acid, mercaptopurine, methyl jasmonate, *N*-methyl-l-glutamic acid, *N*-Methyldioctylamine, *n*-Pentadecylamine, and Salicylic acid. Some of the above metabolites could be significantly linked to special taxons, which indicated that impaired glucose metabolism may be associated with gut micorbiome composition and their metabolites. The potential pathway involved Drug metabolism, Tryptophan metabolism and Citrate cycle.

## Data Availability

The data of this study is available on request.
